# The Potential Cost-Effectiveness of Quadrivalent versus Trivalent Influenza Vaccine in Elderly People and Clinical Risk Groups in the UK: A Lifetime Multi-Cohort Model

**DOI:** 10.1371/journal.pone.0098437

**Published:** 2014-06-06

**Authors:** Laure-Anne Van Bellinghen, Genevieve Meier, Ilse Van Vlaenderen

**Affiliations:** 1 CHESS, Ternat, Belgium; 2 Health Economics, GlaxoSmithKline Vaccines, Wavre, Belgium; Instituto Butantan, Brazil

## Abstract

**Objective:**

To estimate the potential cost-effectiveness of quadrivalent influenza vaccine compared with trivalent influenza vaccine in the UK.

**Methods:**

A lifetime, multi-cohort, static Markov model was constructed, with nine age groups each divided into healthy and at-risk categories. Influenza A and B were accounted for separately. The model was run in one-year cycles for a lifetime (maximum age: 100 years). The analysis was from the perspective of the UK National Health Service. Costs and benefits were discounted at 3.5%. 2010 UK vaccination policy (vaccination of people at risk and those aged ≥65 years) was applied. Herd effect was not included. Inputs were derived from national databases and published sources where possible. The quadrivalent influenza vaccine price was not available when the study was conducted. It was estimated at £6.72,15% above the trivalent vaccine price of £5.85. Sensitivity analyses used an incremental price of up to 50%.

**Results:**

Compared with trivalent influenza vaccine, the quadrivalent influenza vaccine would be expected to reduce the numbers of influenza cases by 1,393,720, medical visits by 439,852 complications by 167,357, hospitalisations for complications by 26,424 and influenza deaths by 16,471. The estimated base case incremental cost-effectiveness ratio (ICER) was £5,299/quality-adjusted life-year (QALY). Sensitivity analyses indicated that the ICER was sensitive to changes in circulation of influenza virus subtypes and vaccine mismatch; all other parameters had little effect. In 96% of simulations the ICER was <£20,000/QALY. Since this analysis was completed, quadrivalent influenza vaccine has become available in the UK at a list price of £9.94. Using this price in the model, the estimated ICER for quadrivalent compared with trivalent vaccination was £27,378/QALY, still within the NICE cost-effectiveness threshold (£20,000-£30,000).

**Conclusions:**

Quadrivalent influenza vaccine could reduce influenza disease burden and would be cost-effective compared with trivalent influenza vaccine in elderly people and clinical risk groups in the UK.

## Introduction

Influenza is a highly infectious acute viral illness. In healthy individuals influenza is generally self-limiting, but complications such as pneumonia may cause serious illness [1]. Children aged <6 months, elderly people (aged ≥65 years), and individuals with conditions such as chronic respiratory or heart disease have an increased risk of influenza complications and serious illness, compared with the general population [1]. The clinical and economic burden of influenza is substantial, estimated at 779,000–1,164,000 general practitioner (GP) consultations, 19,000–31,200 hospital admissions and 18,500–24,800 deaths annually in the UK [2]. In the UK, most cases of influenza tend to occur in a period of 8–10 weeks during the winter (seasonal influenza) [1].

There are three types of influenza virus: A, B and C. In humans, influenza A and influenza B are responsible for most clinical illness. Each can be further subdivided into different subtypes [1]. Influenza A virus strains are categorised by haemagglutinin (H) and neuraminidase (N) antigens, which show small changes from year to year (antigenic drift) and occasional larger changes to a different strain (antigenic shift, resulting in pandemics). Influenza B has two main lineages, Victoria and Yamagata [3]. Influenza B virus seems to cause the same spectrum of disease as influenza A [4], and severe illness can occur with either influenza A or influenza B [5]–[7]. A recent large case-series study suggests that influenza A and B are clinically similar [4]. This study, conducted in persons 6 months of age and older, compared the clinical presentation and risk of radiographic pneumonia and hospital admission among patients with medically attended influenza A and influenza B infections. The investigators identified 901 cases of influenza A and 284 cases of influenza B over four seasons. When data from all four seasons (2004/05–2007/08) were combined, no individual symptom or group of symptoms distinguished influenza A and B infections in children or adults.

Influenza vaccination can protect against infection. At the time this study was initiated, the influenza vaccine recommended in elderly people and clinical risk groups in the UK was inactivated trivalent, i.e. containing two influenza A strains and one influenza B lineage, decided each year according to recommendations from the World Health Organization (WHO) [1]. There is limited cross-protection between the two influenza B lineages, so the effectiveness of each season's vaccine against influenza B depends on correct prediction of the circulating B lineage [3]. Both influenza B lineages have circulated concurrently in recent years, which can limit the effectiveness of the trivalent vaccine against influenza B. In the UK, the vaccine influenza B lineage and the circulating influenza B lineage were at least partially mis-matched in six of the ten influenza seasons from 2000/2001 to 2009/2010 [8]. This phenomenon is not limited to the UK; in the USA, the trivalent vaccine provided little protection against influenza B in five of the ten influenza seasons between 2001 and 2010 [3].

A quadrivalent influenza vaccine including both influenza B lineages could potentially improve protection against influenza B infection and reduce morbidity and mortality due to influenza B disease. An inactivated quadrivalent influenza vaccine has shown improved immunogenicity, compared with trivalent vaccines, in clinical trials in children [9], adults and elderly people [10], [11]. This quadrivalent vaccine (licensed for all individuals 3 years and older) was introduced in the UK in the autumn of 2013, after this study was completed; while available inactivated trivalent vaccines are indicated for individuals as from 6 months old.

The objective of the present study was to estimate the potential cost-effectiveness of inactivated quadrivalent influenza vaccination, compared with inactivated trivalent vaccination, in elderly people and clinical risk groups aged ≥3 years in the UK, which is in agreement with the 2010 UK influenza disease management policy.

At the time we conducted the analysis, UK Department of Health guidance on influenza vaccination (issued in 2010) recommended annual vaccination with inactivated trivalent vaccine of all people aged ≥65 years, workers in healthcare and social care, carers, patients in long-stay care, pregnant women at any stage of pregnancy, and people aged ≥6 months in a clinical risk group (chronic respiratory, heart, liver, kidney or neurological disease, diabetes or immunosuppression) [1].

This policy has recently been amended, with the Joint Committee on Vaccination and Immunisation (JCVI) recommending the extension of the routine annual flu immunisation programme to all children aged two to under 17 years. JCVI advised that all children should be offered a live attenuated trivalent intranasal influenza vaccine unless contra-indicated [12], [13]. The latter vaccine is contra-indicated in patients with immunodeficiency, and should not be used in individuals with severe asthma or active wheezing [14]. In those patients, inactivated trivalent influenza vaccine for children as from 6 months old or inactivated quadrivalent vaccine for children aged three years and older should be offered [15]. Children with immunodeficiency, severe asthma or active wheezing are potential candidates for quadrivalent influenza vaccination and were already included as clinical risk groups in the 2010 guidelines for trivalent inactivated vaccination. As such, these children are covered in our analysis comparing inactivated trivalent vaccination with inactivated quadrivalent vaccination.

## Methods

### Rationale for model design

A structured literature review was conducted to identify published models of influenza in the UK, to assess existing modelling approaches to influenza disease management in different target populations. This review identified 12 UK cost-effectiveness studies (some publications reported more than one study) [16]–[25]. All the identified UK-specific modelling studies used a decision-tree model structure or bootstrap analysis with a time horizon of one annual influenza season. The models thus did not consider the policy effect of repeated annual vaccination in multiple consecutive influenza seasons, and were consequently unable appropriately to incorporate accumulated quality-adjusted life-years (QALYs) gained by different age groups over a lifetime. Only one [24] included the entire population of the UK, including people resident in long-term care facilities. All the others conducted analyses in population age-subgroups (e.g. children, adults or elderly people), without consideration of populations resident in long-term care facilities.

Parameters such as the probability of influenza infection and treatment cost vary between population groups. In particular, although patients aged ≥65 years are commonly categorised as ‘elderly’ and treated as one homogenous group, there is considerable heterogeneity at different ages as natural mortality increases with age [26]. Subdividing the elderly population into several age cohorts approximates the population more closely than a single elderly cohort. To account for these differences, a multi-cohort modelling approach was selected.

A lifetime multi-cohort model was developed, in which cohorts entered the model at different ages and were followed over a lifetime of consecutive influenza seasons. This provides a more direct model of influenza management than a one-year model and allows for appropriate attribution of QALYs over time.

### Model structure

**Figure 1:**
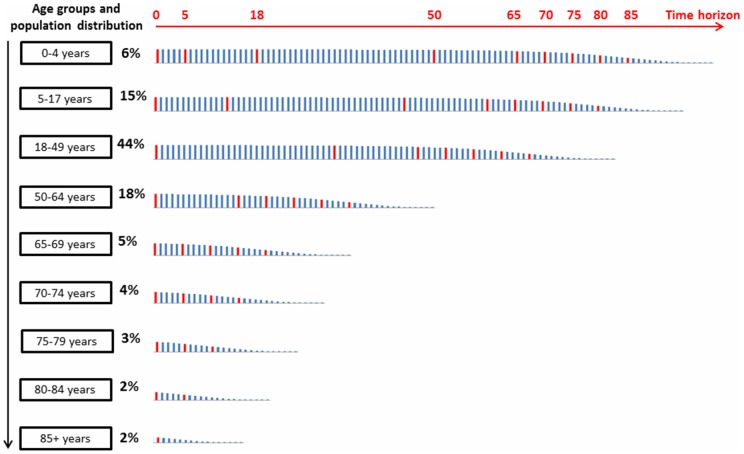
Multi-cohort approach.

We constructed a static lifetime multi-cohort Markov model with a one-year cycle time, reflecting annual winter influenza seasons and applying the UK 2010 guidelines for interventions (vaccination, post-exposure prophylaxis (PEP) and/or antiviral treatment) [1], [27], [28]. Nine age groups were included (0–4, 5–17, 18–49, 50–64, 65–69, 70–74, 75–79, 80–84, ≥85 years), each subdivided into healthy and at-risk. Within the at-risk group we distinguished between those in a clinical risk group (who may receive PEP only if not effectively protected by vaccination) and those resident in long-term care facilities (who may receive PEP regardless of vaccination status). Clinical risk groups include patients with one or more of the following characteristics: chronic respiratory disease; chronic heart disease; chronic renal disease; chronic liver disease; chronic neurological conditions; diabetes mellitus; aged 65 years or older; immunosuppressed (including transplanted patients) [28]. Figure 1 provides a visual representation of the multi-cohort approach. At time zero, the total 2010 UK population is represented in the aforementioned 9 distinct age cohorts according to the UK population distribution [29]. The time horizon of the model was set at 100 years. Once an age cohort reaches the starting age of the next age cohort, the probabilities, costs and effects of the new age group are considered. The youngest age group, i.e. 0–4 years, is followed for a real 100 years while the cohort moves through all age groups. An older age group, for example, 70–74 years, is followed only for 30 years because all individuals within that cohort will have died after that period. Influenza A and B were accounted for separately, to allow the model to evaluate differences in vaccine protection.

In each annual cycle, a number of events could happen. An individual may be vaccinated; receive PEP; become infected with influenza; seek medical advice (from a GP or accident and emergency [A&E] department); medical advice may be followed by antiviral treatment; the influenza virus may be treatment-resistant; influenza-related complications (respiratory or non-respiratory) may develop; an influenza-related complication may lead to hospitalisation or outpatient treatment; an individual may die from influenza or non-influenza-related causes (all-cause mortality). Surviving individuals moved to the next annual cycle. The model process was dichotomised, i.e. at any node with two possible outcomes (e.g. vaccinated or not vaccinated) any change in the probability of one branch was matched by an equal and opposite change in the probability of the other branch.

We assumed that patients with influenza complications who were not hospitalised were treated as outpatients, and that hospitalisation for an influenza complication was preceded by a GP visit [23], [24].

Respiratory complications included bronchitis, pneumonia or upper respiratory tract infection, and non-respiratory complications included cardiac, renal or central nervous system complications, otitis media or gastro-intestinal bleeding [1], [16], [27], [28].

**Figure 2:**
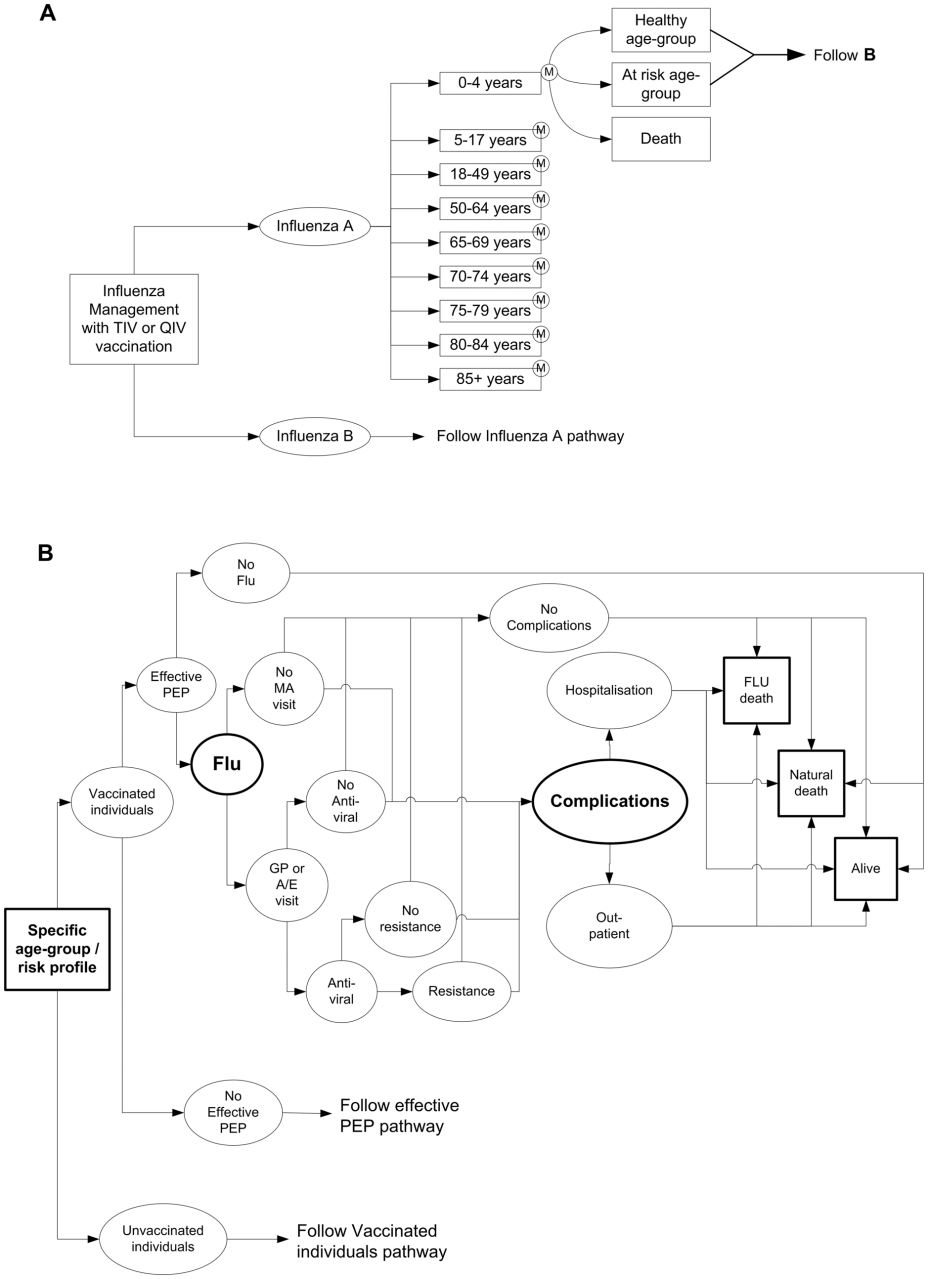
A: Overview of model structure; B: Overview of possible event pathways. A/E, accident and emergency; Alive  =  Healthy or At risk; GP, general practitioner; MA, medical advice; PEP, post-exposure prophylaxis; QIV, quadrivalent vaccine; TIV, trivalent vaccine. In Figure 2A, M in circle  =  Markov node. In Figure 2B, square boxes are start/end points.

Figure 2A shows an overview of the model structure, and Figure 2B shows the various event pathways.

The model operated on the basis of influenza infections (not influenza-like illnesses). It compared trivalent versus quadrivalent influenza vaccination. We assumed similar clinical outcomes for influenza A and B based on the available evidence comparing the clinical impact of different strain types [4]–[7], [30], [31],The main outcome measure was QALYs gained, commonly used in cost-effectiveness analyses. Costs and outcomes were discounted at 3.5% per year, in line with UK guidelines [32]. The analysis was conducted from the perspective of the UK National Health Service (NHS), the national healthcare provider, and thus included only direct medical costs. The model was developed using Microsoft *Excel* 2010.

Herd effect was not included in the model.

### Inputs and assumptions

Most input data used in the model were derived from published economic evaluations of influenza management [16], [23], [24], other published studies [33]–[37] and national databases [38]–[40]. Where data were lacking we took assumptions from published literature if available.

#### Vaccine efficacy and coverage

Vaccine efficacy and coverage data are shown in Table 1.

**Table 1:** Input data for vaccine efficacy and coverage.

Parameter	Age group (years)								
	0–4	5–17	18–49	50–64	65–69	70–74	75–79	80–84	85+
**Vaccine efficacy against influenza A, % ** **[33][34][35]** a **[23]**									
Trivalent and quadrivalent	59.00	59.00	61.00	61.00	58.00	58.00	58.00	58.00	58.00
**Vaccine efficacy against influenza B, % ** **[41]** ** (healthy adults; other age groups assumed)** a									
Trivalent, match	66.00	77.00	77.00	73.00	69.00	69.00	66.00	66.00	66.00
Trivalent, mismatch	44.00	52.00	52.00	49.00	47.00	47.00	44.00	44.00	44.00
Trivalent, average	55.52	65.09	65.09	61.57	58.52	58.52	55.52	55.52	55.52
Quadrivalent	66.00	77.00	77.00	73.00	69.00	69.00	66.00	66.00	66.00
**Vaccine coverage, % ** **[39]**									
Healthy	0	0	0	0	71.19	71.19	71.19	71.19	71.19
At-risk	17.18 b	44.48	44.48	44.48	71.19	71.19	71.19	71.19	71.19

a No difference in input values between healthy and at-risk populations.

b Adjusted for the proportion of the 0–4 age group falling within the product license (i.e. 38.62%).

The average efficacy of the trivalent vaccine against influenza A was estimated from three Cochrane reviews in healthy children [33], healthy adults [34] and elderly people [35]. Vaccine efficacy against influenza A was assumed to be identical for the quadrivalent and trivalent vaccine.

The average efficacy of the trivalent vaccine against vaccine-matched and mismatched B lineages was estimated from a meta-analysis in adults [41] (Table 1). For children and elderly patients, there were no estimates in the literature for trivalent matched or mismatched influenza B efficacy. Inactivated trivalent vaccine efficacy is lower in young children and elderly people than in adults [33], [41], [42] so the adult estimates were reduced and applied to these age groups. Trivalent vaccine has some cross-protection against the mismatched/co-circulating second influenza B lineage [41], [43]. Vaccine efficacy against influenza A and B was assumed to be unaffected by risk status [23].

To model the difference in efficacy against influenza B between the trivalent and quadrivalent vaccines, we assumed that:

- Trivalent vaccine efficacy against influenza B is proportional to the percentage match with circulating influenza B (Table 1). Base-case estimates of the average match between the trivalent vaccine and the circulating influenza B lineage (52.36%) were calculated for 2000–2010 from HPA data [8];
- Vaccine efficacy of quadrivalent vaccine equals the efficacy of trivalent vaccine with optimal matching [9], [10] (Table 1).

The overall impact of efficacy differences between the quadrivalent and trivalent vaccine also depends on the proportion of circulating influenza B within all influenza cases (A+B), which varies by year. Base-case estimates of the average distribution of influenza cases between influenza A (75.16%) and influenza B (24.84%) were calculated for 2000–2010 from HPA data [8].

Based on the calculated 52.36% match between the trivalent vaccine and the circulating influenza B lineage and the 24.84% circulation of influenza B cases, the vaccine efficacy of quadrivalent vaccine against influenza B was estimated to be ∼18% higher compared to the trivalent vaccine across all age groups.

Vaccination coverage according to UK policy (vaccination of people at risk and aged ≥65 years) was calculated from HPA data over the period 2000/2001 to 2008/2009 [39] (Table 1). The children covered in the model reflect the age indication for the GlaxoSmithKline quadrivalent inactivated vaccine (aged ≥3 years).

We assumed that a breakthrough case of influenza following vaccination was not a milder episode compared with no vaccination (no data available) in the base case.

#### Probabilities

The main age-dependent probabilities are summarised in Table 2: probability of influenza infection, medical advice visits, complication, hospitalisation and death due to influenza. For details of the probabilities related to PEP and antiviral treatment and the probabilities of different complication types, see File S1.

**Table 2:** Input data for age-dependent probabilities.

Parameter	Age group (years)								
	0–4	5–17	18–49	50–64	65–69	70–74	75–79	80–84	85+
**Probabilities**									
Symptomatic influenza infection, % [24] a ^,^ b	19.21	19.21	6.55	6.55	6.17	6.17	6.17	6.17	6.17
Seeking medical advice, % [24] c	15.51	15.51	28.19	28.19	32.51	32.51	32.51	32.51	32.51
**Probability of complication without antiviral treatment, % ** **[36]** d									
Healthy	14.05	14.05	7.61	7.95	10.34	10.34	10.34	10.34	10.34
At-risk	18.29	18.29	12.32	12.59	13.76	13.76	13.76	13.76	13.76
Relative risk of complication after antiviral treatment, at-risk only % [23]	69.45	61.87	47.77	47.84	48.18	48.18	48.18	48.18	48.18
**Probability of hospitalisation following any complication, % ** **[23]**									
Healthy	10.87	10.87	10.87	10.87	15.79	15.79	15.79	15.79	15.79
At risk	15.79	15.79	15.79	15.79	15.79	15.79	15.79	15.79	15.79
**Probability of death following hospitalisation or outpatient treatment for complication, % ** **[36]** e									
Healthy	0	0	0.41	0.96	11.21	11.21	11.21	11.21	11.21
At risk	0.15	0.15	0.34	1.64	12.18	12.18	12.18	12.18	12.18
**Probability of death from uncomplicated influenza, % (assumption)**									
Healthy and At risk	0	0	0	0	0	0	0	0	0

a No difference in input values between healthy and at-risk populations.

b Identical for influenza A and B.

c 97.0% present to a GP and 3.0% to A & E, in all age groups [23].

d No difference between cases presenting to GP or A & E, or not seeking medical advice.

e Risk of death assumed to be the same for all complications.

A&E, accident and emergency; GP, general practitioner.

The probability of moving from healthy to at-risk in each cycle was assumed to be independent of influenza exposure and vaccination status, and was calculated from all-cause mortality data and the age distribution of the at-risk population (defined as described above) (see File S1).

Demographic data (mid-year 2010 estimates) [29] and all-cause mortality data [44], [45] were obtained from the Office for National Statistics (ONS), and the proportion of each age group categorised as at-risk from published sources [24], [46]. All-cause mortality in the at-risk population was assumed to be ten times the all-cause mortality in the healthy population [47] (see File S1).

We assumed that at-risk individuals (defined as described above) remained at risk for their remaining lifetimes.

#### Costs

The reference year for costs was 2010. The trivalent vaccine price was £5.85, calculated as the average cost of 11 trivalent vaccines in the British National Formulary (BNF) in 2011 [48] (no adjustment required to the reference year of 2010 as the BNF price change between 2010 and 2011 was minimal). The quadrivalent vaccine price was assumed to be 15% higher (£6.72), as no information on the quadrivalent vaccine price was available at the time of the study. The administration cost was £36.00, the cost of a GP visit in 2010 [40]. For people aged ≥65 years we assumed that vaccination would take place as part of a regular check-up visit or visit for chronic prescriptions refill, and thus no additional cost of a GP visit was incurred for these people. The costs of GP and A&E visits, antibiotics and outpatient treatment of complications are summarised in Table 3. Hospitalisation costs were obtained from NHS reference costs [38] (Table 3). Neuraminidase medication costs (for treatment and PEP) were estimated from the BNF [48] (Table 3). The costs of over-the-counter medication were not included because such costs are borne by the patient and the present analysis was conducted from the NHS perspective.

**Table 3:** Input data for costs.

Parameter	Age group (years)								
	0–4	5–17	18–49	50–64	65–69	70–74	75–79	80–84	85+
**Costs, £**									
Cost of a GP visit [40]	36.00	36.00	36.00	36.00	36.00	36.00	36.00	36.00	36.00
Cost of A&E visit [38]	128.00	128.00	128.00	128.00	128.00	128.00	128.00	128.00	128.00
Cost of outpatient treatment [40]	112.50	112.50	112.50	112.50	112.50	112.50	112.50	112.50	112.50
Cost of antibiotics [23]	0.69	0.65	0.98	0.98	1.28	1.28	1.28	1.28	1.28
Cost of neuraminidase inhibitor medication (for treatment and PEP) [48]	15.41	15.41	15.89	15.89	15.89	15.89	15.89	15.89	15.89

Hospitalisation cost a and average length of stay for different types of complications [38]									
**Bronchitis hospitalisation**									
Cost	£2,239.22	£2,239.22	£2,239.22	£2,239.22	£2,239.22	£2,239.22	£2,239.22	£2,239.22	£2,239.22
LOS	5.84	5.84	5.84	5.84	5.84	5.84	5.84	5.84	5.84
**Pneumonia hospitalisation**									
Cost	£2,732.47	£2,732.47	£2,732.47	£2,732.47	£2,732.47	£2,732.47	£2,732.47	£2,732.47	£2,732.47
LOS	7.53	7.53	7.53	7.53	7.53	7.53	7.53	7.53	7.53
**URTI hospitalisation**									
Cost	£1,500.63	£1,500.63	£1,500.63	£1,500.63	£1,500.63	£1,500.63	£1,500.63	£1,500.63	£1,500.63
LOS	2.18	2.18	2.18	2.18	2.18	2.18	2.18	2.18	2.18
**Hospitalisation for cardiac complications**									
Cost	£2,552.38	£2,552.38	£2,552.38	£2,552.38	£2,552.38	£2,552.38	£2,552.38	£2,552.38	£2,552.38
LOS	6.51	6.51	6.51	6.51	6.51	6.51	6.51	6.51	6.51
**Hospitalisation for renal complications**									
Cost	£2,189.80	£2,189.80	£2,189.80	£2,189.80	£2,189.80	£2,189.80	£2,189.80	£2,189.80	£2,189.80
LOS	4.98	4.98	4.98	4.98	4.98	4.98	4.98	4.98	4.98
**CNS hospitalisation**									
Cost	£3,226.89	£3,226.89	£3,226.89	£3,226.89	£3,226.89	£3,226.89	£3,226.89	£3,226.89	£3,226.89
LOS	9.19	9.19	9.19	9.19	9.19	9.19	9.19	9.19	9.19
**OM hospitalisation**									
Cost	£1,936.20	£1,936.20	£1,936.20	£1,936.20	£1,936.20	£1,936.20	£1,936.20	£1,936.20	£1,936.20
LOS	3.68	3.68	3.68	3.68	3.68	3.68	3.68	3.68	3.68
**Hospitalisation for GI bleeding and other complications**									
Cost	£1,519.88	£1,519.88	£1,936.61	£1,936.61	£1,890.79	£1,890.79	£1,890.79	£1,890.79	£1,890.79
LOS	24.32	24.32	6.06	6.06	8.07	8.07	8.07	8.07	8.07

a 4.9% of hospitalised patients are treated in intensive care [24], and this cost was included in the cost of hospitalization.

A&E, accident and emergency; CNS, central nervous system; GI, gastrointestinal; GP, general practitioner; LOS, length of stay; OM, otitis media; PEP, post-exposure prophylaxis; URTI, upper respiratory tract infection.

We assumed that there was no impact of adverse effects of vaccination, PEP or neuraminidase antiviral treatment on costs [23], [24].

#### Utilities

Utility data are summarised in Table 4. Disutilities were derived from EuroQoL data reported for uncomplicated influenza [19], and from assumptions presented in a previous publication [37] for hospitalised cases. We assumed that there was no impact of adverse effects of vaccination, PEP or neuraminidase antiviral treatment on utilities [23], [24].

**Table 4:** Input data for utilities.

Parameter	Age group (years)								
	0–4	5–17	18–49	50–64	65–69	70–74	75–79	80–84	85+
**Utilities**									
Baseline utility [23] a	0.94	0.94	0.91	0.82	0.78	0.78	0.73	0.73	0.73
Disutility for influenza [19] b	−0.88	−0.88	−0.88	−0.88	−0.88	−0.88	−0.88	−0.88	−0.88
Disutility for influenza complication treated in hospital [37] c	−0.98	−0.98	−0.98	−0.98	−0.98	−0.98	−0.98	−0.98	−0.98

a No difference in input values between healthy and at-risk populations.

b Duration 7.5 days without antiviral treatment, 5.0 days with antiviral treatment.

c Duration 5.4 days. Outpatient-treated complications assumed to have the same duration as hospitalised complications, and the same disutility as influenza. Disutilities and durations are identical for all types of complications.

### Sensitivity analyses

One-way and probabilistic sensitivity analyses evaluated the effect on the model results of uncertainty in the input data. For the one-way sensitivity analysis, each parameter was varied from its base-case value within a range/distribution.

The probabilistic sensitivity analysis was performed using Monte Carlo simulation with 1,000 iterations, each selecting the input parameter values from a probability distribution. The data values, ranges of the parameters for the one-way sensitivity analysis and the probability distributions for the probabilistic sensitivity analysis are shown in File S1 for the parameters with the highest impact on the incremental cost-effectiveness ratio (ICER). All parameters were included in the probabilistic sensitivity analysis except for a few that were considered fixed (population distribution, vaccine efficacy against influenza A, probability of death from uncomplicated influenza, trivalent and quadrivalent vaccine cost, vaccine administration cost, PEP and antiviral treatment cost, GP visit cost, antibiotic cost, outpatient treatment cost).

### Model validation

The model structure was validated by checking against the International Society for Pharmacoeconomics and Outcomes Research (ISPOR) modelling study guidance criteria [49]. Technical model validation was performed by reproducing one cohort of the model in TreeAge Pro 2011.

## Results

### Base case

Table 5 shows the estimated number of individuals receiving vaccination and/or PEP treatment, the lifetime disease burden (i.e. the estimated number [accumulated over lifetime] of influenza cases, medically attended cases, cases receiving antiviral treatment, complications, hospitalisations outpatient treatment for complications and deaths) with trivalent or quadrivalent influenza vaccination in the base case, together with estimates of QALYs and life-years gained, costs and the ICER for the quadrivalent vaccine compared with the trivalent vaccine, under the 2010 UK guidance on management of influenza. The estimated numbers vaccinated and receiving PEP are greater with quadrivalent vaccination than with trivalent vaccination because this is a lifetime model. As more people survive with quadrivalent vaccination, there are more people in the quadrivalent vaccine group than the trivalent vaccine group who are at risk of influenza infection and may therefore receive PEP and/or vaccination in each successive influenza season. The model accumulates these data over the lifetime of the multi-cohort (from birth to 100 years for the youngest age cohort, thus from 2010 to 2110 in this age group; from 85 to 100 years for the oldest age cohort, thus from 2010 to 2025 in this age group), and so the cumulative number of vaccinations and PEP treatments is higher with quadrivalent vaccination.

**Table 5:** Base case results.

	Trivalent vaccine	Quadrivalent vaccine	Difference (Quadrivalent minus Trivalent)
**Lifetime disease burden**			
Number vaccinated	840,265,354	840,385,839	120,485
Number receiving PEP	6,500,388	6,500,874	486
Number of influenza cases	200,640,122	199,246,402	–1,393,720
Number seeking medical treatment for uncomplicated influenza	53,636,585	53,196,733	–439,852
Number receiving antiviral treatment	1,255,050	1,223,977	–31,073
Number with influenza complications	19,847,051	19,679,693	–167,357
Number of hospitalisations for complications	2,453,715	2,427,290	–26,424
Number of outpatients treated for complications	17,393,336	17,252,403	–140,933
Number of influenza deaths a	584,986	568,515	–16,471
**Health outcomes (discounted)**			
QALYs	1,190,979,257	1,191,015,259	36,002
Life-years	1,375,979,430	1,376,016,515	37,085
**Costs (discounted), £**			
Vaccination	3,281,765,398	3,498,638,907	216,873,510
PEP	152,842,191	152,847,904	5,712
Treatment of uncomplicated influenza	992,298,230	986,901,062	–5,397,168
Hospitalisation for complications	2,147,114,980	2,131,326,102	–15,788,878
Outpatient treatment of complications	1,004,651,24	999,736,884	–4,914,359
**Total**	**7,578,672,042**	**7,769,450,859**	**190,778,818**
**Incremental cost-effectiveness ratio, quadrivalent vaccine versus trivalent vaccine (discounted)**			
£/QALY gained	-	-	5,299
£/life-year gained	-	-	5,144

PEP, post-exposure prophylaxis; QALY, quality-adjusted life-year.

a All deaths due to complications, as it was assumed that mortality from uncomplicated influenza was 0.

Quadrivalent vaccination would be expected to reduce the disease burden of influenza further, compared with trivalent vaccination (Table 5). For example, the expected reduction in the number of cases would be 1,393,720. The number of QALYs would be expected to increase by 36,002 (Table 5). The total cost would also be expected to increase by over £190 million (Table 5). This reflects the greater cost of the quadrivalent vaccine compared with the trivalent vaccine and the higher survival with quadrivalent vaccine, which would increase the number of people who survive and remain eligible for further influenza vaccinations over their lifetime. These increased costs would be partially offset by reductions in the costs of treating influenza, particularly hospitalisations for complications. With a quadrivalent influenza vaccine price of £6.72 per dose, the estimated discounted ICER for the quadrivalent vaccine compared with the trivalent vaccine was £5,299 per QALY gained.

### Lifetime results versus one-year results

**Figure 3:**
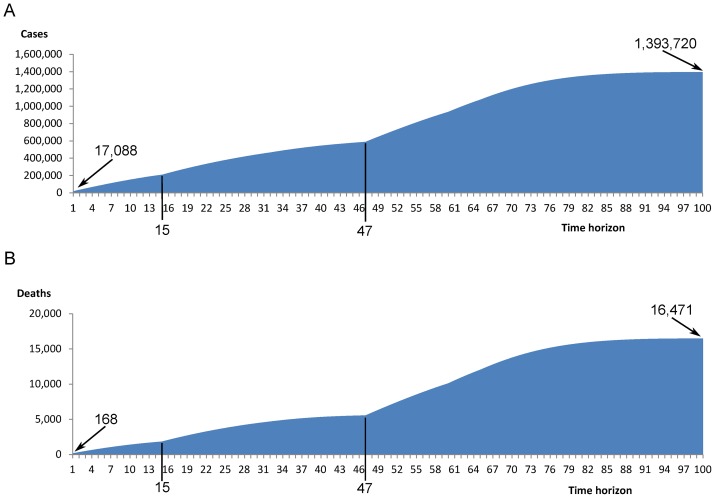
Influenza cases and deaths averted by quadrivalent vaccination over time. A: Cumulative number of influenza cases expected to be averted by quadrivalent vaccination compared with trivalent vaccination in each year of the model. B: Cumulative number of influenza deaths expected to be averted by quadrivalent vaccination compared with trivalent vaccination in each year of the model.

Figure 3A and Figure 3B show the number of influenza cases and deaths, respectively, predicted to be averted by quadrivalent vaccination compared with trivalent vaccination in each year of the model. In the first year, individuals of all nine age groups enter the model according to the population age distribution. In the first year, quadrivalent vaccination would be expected to avert 17,088 additional influenza cases and 168 influenza deaths. As the cohorts age and move through the model, the number of cases and deaths averted each year is added to the total. By year 100, the accumulated number of cases averted would be 1,393,720 and the number of deaths averted would be 16,471. There are two inflection points in the curves, reflecting age-related changes in the percentages of healthy individuals who receive influenza vaccination; this percentage increases from 0% to 71.19% in the group aged 65+ years. The first occurs at 15 years when the individuals who entered the model at age 50 years (representing 44% of the population) reach the age of 65 years, and the second occurs at 47 years when the individuals who entered the model at age 18 years (representing 18% of the population) reach the age of 65 years.

Table 6 shows the estimated number of vaccinated individuals, influenza cases, medically attended cases, complications, hospitalisations and deaths with trivalent or quadrivalent influenza vaccine in the first year of vaccination. Unlike the cumulative lifetime results in Table 5, there is no difference in the number of individuals vaccinated, because the results in Table 6 cover only a single year and so there is no scope for differential survival to affect the number of individuals in the cohort under different vaccination strategies.

**Table 6:** Results in the first year.

	Trivalent vaccine	Quadrivalent vaccine	Difference (Quadrivalent minus Trivalent)
Number vaccinated	9,564,536	9,564,536	0
Number of influenza cases	5,314,312	5,297,224	–17,088
Number seeking medical treatment for uncomplicated influenza	1,199.856	1,194,735	–5,121
Number with influenza complications	595,448	593,316	–2,132
Number of hospitalisations for complications	69,067	68,731	–337
Number of influenza deaths a	6,427	6,259	–168

a All deaths due to complications, as it was assumed that mortality from uncomplicated influenza was 0.

### Sensitivity analyses

The one-way sensitivity analysis showed that only two parameters, the distribution of influenza A and B and the degree of vaccine mismatch, had large effects on the estimated ICER. This is to be expected, since the difference between the quadrivalent and trivalent vaccines is the efficacy against influenza B. In the case of 99.6% circulation of influenza A (i.e. almost no influenza B circulation), the ICER reaches £373,000 per QALY gained. In the case of 99.0% matching of TIV with circulating influenza B virus, the ICER reaches £286,000 per QALY gained. On the other hand, in the case of 30% circulation of influenza B (corresponding with the lowest circulation of influenza A virus during the influenza seasons 2000/2001 to 2009/2010), the ICER reaches £1,400 per QALY gained and in case of 0% matching, the ICER reaches £2,149 per QALY gained.

Another parameter that had an impact on the ICER was the price of the quadrivalent vaccine. A 50% increase in price of the quadrivalent vaccine in all age groups (to £10.08) results in an ICER of £28,443 per QALY gained. No other parameter had a substantial effect on the ICER.

**Figure 4:**
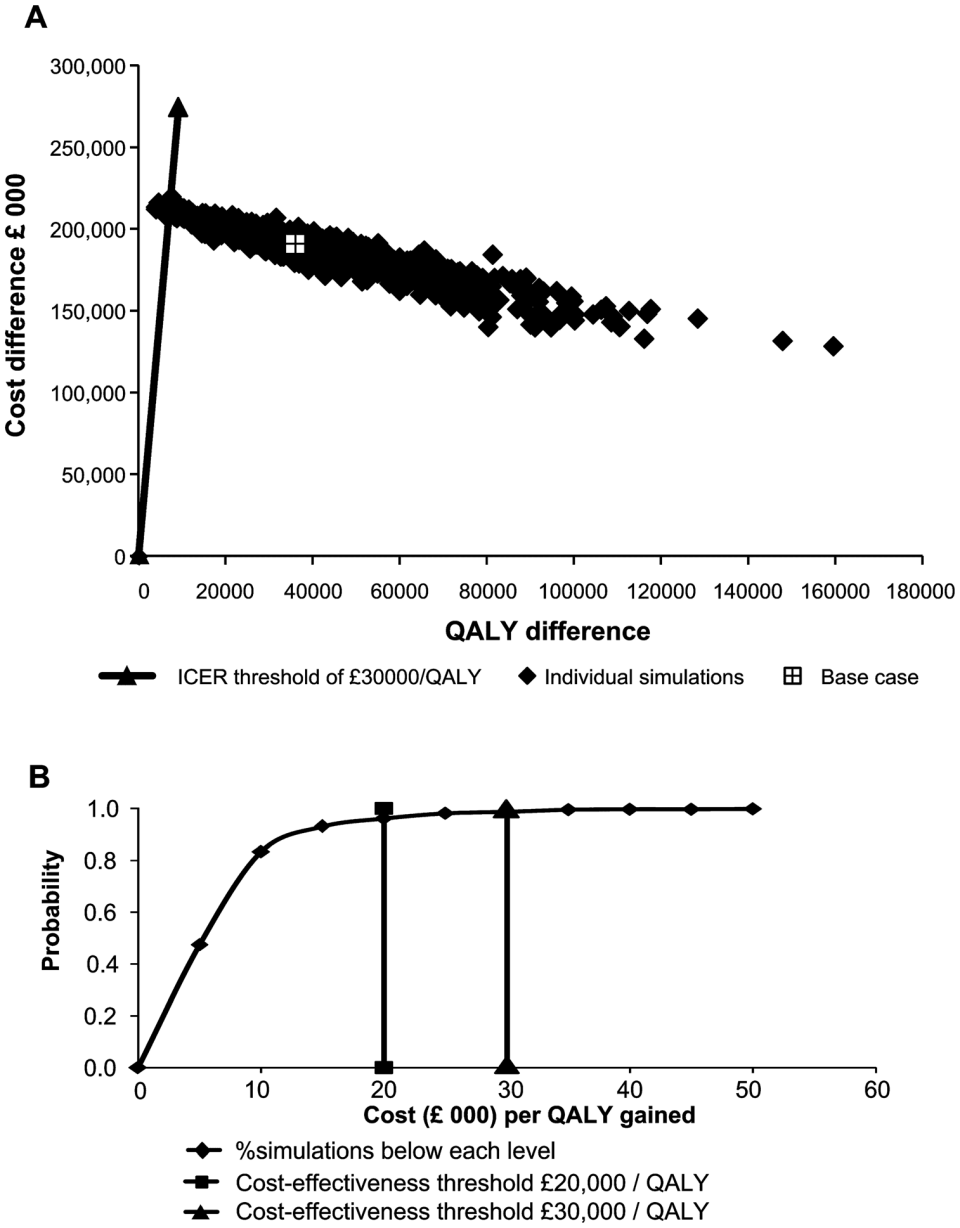
Probabilistic sensitivity analysis. A: cost-effectiveness plane; B: cost-effectiveness acceptability curve. ICER, incremental cost-effectiveness ratio; QALY, quality-adjusted life-year.

Figure 4A shows the cost-effectiveness plane from the probabilistic sensitivity analysis The cost-effectiveness acceptability curve is shown in Figure 4B. In total, 96% of the simulations were below a threshold of £20,000/QALY, and 99% were below £30,000/QALY.

## Discussion

This analysis used a lifetime, multi-cohort Markov model to compare the potential effects of quadrivalent and trivalent influenza vaccines on the disease burden of influenza in the UK from the perspective of the NHS. Our base-case results indicated that the quadrivalent influenza vaccine would be expected to result in substantial health benefits, further reducing the number of symptomatic influenza cases by 1,393,720, the number of medical visits by 439,852, the number of complications by 167,357, the number of hospitalisations for complications by 26,424 and the number of deaths by 16,471, compared with a trivalent vaccine. The estimated reduction in influenza cases with the quadrivalent vaccine would also reduce the costs of treating influenza, partially offsetting the increased costs of quadrivalent vaccination compared with the trivalent vaccine. Overall, the quadrivalent vaccine was estimated to be a cost-effective intervention compared with the trivalent vaccine, with an ICER estimated at £5,299/QALY. This is below the threshold range of £20,000/QALY to £30,000/QALY considered cost-effective by the National Institute for Health and Clinical Excellence (NICE) [50].

At the time of the analysis, the price of the quadrivalent vaccine was not available and so we had to make an assumption. Since we completed the study, quadrivalent influenza vaccine has been made available in the UK at a list price of £9.94. This is considerably higher than the price assumption used in our analysis (which was £6.72, 15% higher than trivalent vaccines). When the list price of £9.94 was applied to our model, the ICER for quadrivalent vaccination compared with trivalent vaccination was £27,378/QALY. This is still within the threshold range of £20,000/QALY to £30,000/QALY considered cost-effective by NICE [50].

Our results are consistent with findings from a study in the US, which indicated that quadrivalent influenza vaccine would be expected to reduce influenza cases, hospitalisations and deaths, compared with a trivalent vaccine [51].

Previous economic evaluations of influenza vaccination have typically considered a one-year time horizon [16], [23], [24]. This approach can estimate lifetime benefits only by artificially extrapolating the one-year results to estimate the number of life-years gained from a reduction in influenza deaths. Furthermore, a one-year model evaluates the cost-effectiveness of a single vaccination, which does not match the current health policy of vaccination repeated each annual influenza season. A lifetime model such as ours can follow a cohort of patients over a lifetime of influenza seasons with repeated vaccination and/or other interventions (e.g. PEP) as appropriate each year, and thus more closely reflects health policy in the real world. A lifetime cohort model is better suited than a one-year model to answering research questions about the cost-effectiveness of a particular vaccination policy when applied to today's eligible population cohorts, who will then age over time. However, because of the difference in modelling approach, our results are not directly comparable with previously published results from one-year models.

We chose to use a multi-cohort model to reflect population heterogeneity. Different age groups may vary in their probability of infection, baseline utility, mortality risk and other factors. We divided the elderly population (aged ≥65 years) into 5-year age groups. These age groups are smaller than the age ranges we used for adults or adolescents, in order to capture age-dependent variations in influenza risk, complications, disease management etc. However, this capacity for heterogeneity was often not reflected in our data inputs, as detailed age-specific data proved difficult to find. More research in this area would be valuable to provide a more detailed picture of influenza in elderly individuals.

Our model has a number of limitations. First, recommendations for influenza management continue to be changed and updated, and even since the development of this model several significant policy changes have occurred in the management of influenza in the UK. As stated in the introduction, the UK influenza vaccination guidelines have been extended to include live attenuated intranasal influenza vaccination in all children aged two to under 17 years, unless contra-indicated. Children with immunodeficiency, severe asthma or active wheezing are potential candidates for quadrivalent influenza vaccination and are captured in the risk groups in the present model. As such, the new extension policy does not affect our analysis, which is intended to assist in making policy decisions around the use of inactivated quadrivalent versus inactivated trivalent influenza vaccination in the UK in elderly people and clinical risk groups. PEP and antiviral treatment of influenza with oseltamivir and zanamivir were modelled according to 2010 UK guidelines [27], [28]. Since we conducted the analysis, new guidance has been issued by Public Health England [52]. Current recommendations make wider use of post-exposure prophylaxis and antiviral treatment than suggested in this model. As these recommendations changed after our study was finalised they are not included in the present analysis. However, we assessed the potential impact of extending the use of PEP and antiviral treatment, by considering the same proportion of PEP and treatment for healthy individuals as for at-risk individuals in a scenario analysis. This scenario does not completely match the new recommendations but is indicative of the magnitude and direction of the impact on the ICER. At the base-case vaccine price of £6.72, the new ICER for quadrivalent vaccination compared with trivalent vaccination was £5.433.87, a 2.5% difference compared to the base case analysis considering the 2010 guidelines. This is a relatively minor change, indicating that updating the model with the new recommendations regarding PEP and antiviral use is unlikely to have a substantial impact on the overall results. Models will need to be frequently updated and amended to take account of changes in recommended practice.

Second, it is a static model and thus cannot fully account for herd effects. A dynamic model would be better able to evaluate herd effect.

Third, seasonal variations in circulating influenza virus subtypes and vaccine mismatch are not incorporated directly. They are allowed for in the base case by taking a ten-year average from historical data, and the probabilistic sensitivity analysis also takes into account seasonal variability by applying a statistical distribution to the calculated vaccine efficacy. This approach is an approximation, and relies on an implicit assumption that the ten years of historical data used to derive the estimates in the model will be appropriate for the 100-year time horizon of the model. This limitation in relation to the unpredictability of the influenza virus also applies to analyses using one-year models. Although in theory a one-year model could model the exact influenza virus subtypes in circulation in any one year, and could then be re-run with different data to model the virus circulation in a different year, in practice models are unlikely to be updated with new virus circulation data in every year. Furthermore, any decisions made on the basis of a one-year model are likely to be implemented for several years at a time, and are unlikely to be changed every year to reflect differences in virus circulation. It should also be noted that this analysis considers only seasonal influenza vaccination, which is routinely repeated at each annual influenza season as the continual antigenic drift in the influenza virus alters the strains circulating in each influenza season. It does not address the issue of influenza pandemics resulting from occasional antigenic shifts in the influenza virus. Because pandemics occur occasionally, annual seasonal vaccination is not relevant to their management.

Fourth, our model did not include chronic diseases or rehabilitation costs associated with influenza infections [53], [54], and thus potentially underestimated the benefit of quadrivalent influenza vaccination.

Fifth, there were limitations in the data available to populate the model. As well as the lack of detailed age-specific data in elderly individuals mentioned above, there was often a lack of data on differences between healthy and at-risk individuals, e.g. for vaccine efficacy, probability of influenza infection and likelihood of seeking medical advice. Vaccine efficacy data against influenza B were available only from a meta-analysis in healthy adults, and therefore efficacy in children and elderly people had to be assumed. Similarly, although efficacy data for the quadrivalent vaccine in children were published after we had conducted our analysis [55], this trial did not compare the quadrivalent vaccine with the trivalent vaccine and thus was not suitable for the comparison conducted here. Although data on disutility values have been reported [16], these were not patient-reported disutility values and were applied to a 21-day period, rather than for the duration of the influenza episode in days as required by our model. Therefore, the disutility data in the model were taken from a single study in elderly patients (identified as part of a literature review [24]), where disutilities were stratified by severity of influenza episodes [37]. These estimates were varied in the sensitivity analysis to account for uncertainty. Moreover, due to a lack of data, no difference was implemented in disutilities or durations with regard to different complications, which could under- or over-estimate the impact of some complications. Further research would be valuable to explore the impact of mild and severe influenza, with and without complications, on utility scores across a range of age groups.

We evaluated the two vaccines from the perspective of the NHS, and thus our analysis did not take into account indirect costs resulting from time lost from work due to influenza. As the quadrivalent vaccine was estimated to prevent more cases of influenza than the trivalent vaccine, due to improved protection against influenza B, excluding reductions in indirect costs could have underestimated the potential benefits of the quadrivalent vaccine.

In conclusion, this lifetime economic evaluation of quadrivalent compared with trivalent influenza vaccines in elderly people and clinical risk groups in the UK, modelled using a multi-cohort Markov model, estimated that quadrivalent influenza vaccination could further reduce influenza cases, complications, hospitalisations and deaths compared with a trivalent vaccine. A 25% circulation of influenza B and a 52% matching of the B-strain in the quadrivalent influenza vaccine with the B-strain in circulation was considered in the model (average data of ten influenza seasons from 2000/2001 to 2009/2010), leading to a vaccine efficacy of the quadrivalent vaccine against influenza B estimated at ∼18% higher compared to the trivalent vaccine across all age groups. Based on these values and using the base case quadrivalent vaccine price of £6.72 and the recent list price of £9.94, the quadrivalent vaccine was estimated to be cost-effective compared with the trivalent vaccine, with an ICER of £5,299/QALY and £27,378/QALY, respectively. The benefit of quadrivalent vaccine will vary annually depending on the match between the trivalent vaccine and the circulating influenza B lineage.

## Supporting Information

File S1 **Supplementary Material.**
(DOC)
