# Supplementary material for: The Potential Cost-Effectiveness of Quadrivalent versus Trivalent Influenza Vaccine in Elderly People and Clinical Risk Groups in the UK: A Lifetime Multi-Cohort Model
Source: PLoS One. 2014 Jun 6;9(6):e98437. doi: 10.1371/journal.pone.0098437 (PMC4048201; doi:10.1371/journal.pone.0098437)
Supplement: File S1 — Supplementary Material. (DOC) [file pone.0098437.s001.doc]

**The potential cost-effectiveness of quadrivalent versus trivalent influenza vaccine in elderly people and clinical risk groups in the UK: a lifetime multi-cohort model**

**Supplementary Material**

Laure-Anne Van Bellinghen MSc^1^

Genevieve Meier BPharm, MSc^2^

Ilse Van Vlaenderen DVM, MSc^1^

^1^CHESS, Kerkstraat 27, 1742 Ternat, Belgium.

^2^Health Economics, GlaxoSmithKline Vaccines, Avenue Fleming 20, 1300 Wavre, Belgium.

**Probabilities of receiving post-exposure prophylaxis (PEP) [1]**

| Years | 0-4 | 5-17 | 18-49 | 50-64 | 65-69 | 70-74 | 75-79 | 80-84 | 85+ |
| --- | --- | --- | --- | --- | --- | --- | --- | --- | --- |
| **CAR without prior vac*** | 19.98% | 19.98% | 2.10% | 2.10% | 1.36% | 1.36% | 1.36% | 1.36% | 1.36% |
| **CAR with prior vac*** | 0% | 0% | 0% | 0% | 0% | 0% | 0% | 0% | 0% |
| **Residential without prior vac*** | 19.98% | 19.98% | 2.10% | 2.10% | 1.36% | 1.36% | 1.36% | 1.36% | 1.36% |
| **Residential with prior vac*** | 19.98% | 19.98% | 2.10% | 2.10% | 1.36% | 1.36% | 1.36% | 1.36% | 1.36% |

*vac = vaccination; *CAR = clinical at-risk

**Relative risk of influenza after PEP in at-risk group [1]**

| Years | 0-4 | 5-17 | 18-49 | 50-64 | 65-69 | 70-74 | 75-79 | 80-84 | 85+ |
| --- | --- | --- | --- | --- | --- | --- | --- | --- | --- |
| **At Risk** | 42.15% | 35.37% | 22.62% | 22.62% | 22.47% | 22.47% | 22.47% | 22.47% | 22.47% |

**Probabilities of antiviral treatment after seeking medical advice in at-risk group [1]**

| Years | 0-4 | 5-17 | 18-49 | 50-64 | 65-69 | 70-74 | 75-79 | 80-84 | 85+ |
| --- | --- | --- | --- | --- | --- | --- | --- | --- | --- |
| **At Risk** | 52.00% | 52.00% | 16.00% | 16.00% | 11.00% | 11.00% | 11.00% | 11.00% | 11.00% |

**Probabilities of resistance to PEP and neuraminidase inhibitors [2]**

| Years | 0-4 | 5-17 | 18-49 | 50-64 | 65-69 | 70-74 | 75-79 | 80-84 | 85+ |
| --- | --- | --- | --- | --- | --- | --- | --- | --- | --- |
| **At Risk** | 5.03% | 5.03% | 1.77% | 1.77% | 1.77% | 1.77% | 1.77% | 1.77% | 1.77% |

**Probabilities of respiratory complications, given any type of complication is present [1]**

| Years | 0-4 | 5-17 | 18-49 | 50-64 | 65-69 | 70-74 | 75-79 | 80-84 | 85+ |
| --- | --- | --- | --- | --- | --- | --- | --- | --- | --- |
| **Healthy** | 70.08% | 70.08% | 86.52% | 86.52% | 86.50% | 86.50% | 86.50% | 86.50% | 86.50% |
| **At Risk** | 76.51% | 76.51% | 89.41% | 89.41% | 82.60% | 82.60% | 82.60% | 82.60% | 82.60% |

**Probabilities of bronchitis, pneumonia and upper respiratory tract infection in patients with respiratory complications [1] [3]**

| Years | 0-4 | 5-17 | 18-49 | 50-64 | 65-69 | 70-74 | 75-79 | 80-84 | 85+ |
| --- | --- | --- | --- | --- | --- | --- | --- | --- | --- |
| **Probability of bronchitis complication** | | | | | | | | | |
| **Healthy** | 6.65% | 6.65% | 18.75% | 18.75% | 33.29% | 33.29% | 33.29% | 33.29% | 33.29% |
| **At Risk** | 4.03% | 4.03% | 19.05% | 19.05% | 33.91% | 33.91% | 33.91% | 33.91% | 33.91% |
| **Probability of pneumonia complication** | | | | | | | | | |
| **Healthy** | 1.71% | 1.71% | 4.20% | 4.20% | 12.93% | 12.93% | 12.93% | 12.93% | 12.93% |
| **At Risk** | 1.73% | 1.73% | 3.19% | 3.19% | 12.85% | 12.85% | 12.85% | 12.85% | 12.85% |
| **Probability of URTI complication** | | | | | | | | | |
| **Healthy** | 91.64% | 91.64% | 77.04% | 77.04% | 53.78% | 53.78% | 53.78% | 53.78% | 53.78% |
| **At Risk** | 94.24% | 94.24% | 77.75% | 77.75% | 53.25% | 53.25% | 53.25% | 53.25% | 53.25% |

**Probabilities of cardiac, renal, or central nervous system complications, otitis media and gastrointestinal bleeding in patients with non-respiratory complications [1] [3]**

| Years | 0-4 | 5-17 | 18-49 | 50-64 | 65-69 | 70-74 | 75-79 | 80-84 | 85+ |
| --- | --- | --- | --- | --- | --- | --- | --- | --- | --- |
| **Probability of cardiac complication** | | | | | | | | | |
| **Healthy** | 0.00% | 0.00% | 1.48% | 1.48% | 7.03% | 7.03% | 7.03% | 7.03% | 7.03% |
| **At Risk** | 0.00% | 0.00% | 12.61% | 12.61% | 37.11% | 37.11% | 37.11% | 37.11% | 37.11% |
| **Probability of renal complication** | | | | | | | | | |
| **Healthy** | 0.28% | 0.28% | 0.91% | 0.91% | 3.91% | 3.91% | 3.91% | 3.91% | 3.91% |
| **At Risk** | 0.00% | 0.00% | 2.17% | 2.17% | 7.55% | 7.55% | 7.55% | 7.55% | 7.55% |
| **Probability of CNS complication** | | | | | | | | | |
| **Healthy** | 2.34% | 2.34% | 10.82% | 10.82% | 16.41% | 16.41% | 16.41% | 16.41% | 16.41% |
| **At Risk** | 0.00% | 0.00% | 6.52% | 6.52% | 14.47% | 14.47% | 14.47% | 14.47% | 14.47% |
| **Probability of OM complication** | | | | | | | | | |
| **Healthy** | 94.34% | 94.34% | 62.41% | 62.41% | 16.41% | 16.41% | 16.41% | 16.41% | 16.41% |
| **At Risk** | 95.63% | 95.63% | 47.83% | 47.83% | 6.92% | 6.92% | 6.92% | 6.92% | 6.92% |
| **Probability of GI bleeding** | | | | | | | | | |
| **Healthy** | 3.03% | 3.03% | 24.37% | 24.37% | 56.25% | 56.25% | 56.25% | 56.25% | 56.25% |
| **At Risk** | 4.38% | 4.38% | 30.87% | 30.87% | 33.96% | 33.96% | 33.96% | 33.96% | 33.96% |

**Annual probabilities for moving from healthy to at-risk [4-8]**

| Age | Rate | Age | Rate | Age | Rate | Age | Rate | Age | Rate |
| --- | --- | --- | --- | --- | --- | --- | --- | --- | --- |
| **0** | 0.00168 |  |  |  |  |  |  |  |  |
| **1** | 0.00012 | **21** | 0.00021 | **41** | 0.00064 | **61** | 0.00476 | **81** | 0.05018 |
| **2** | 0.00007 | **22** | 0.00020 | **42** | 0.00068 | **62** | 0.00514 | **82** | 0.05566 |
| **3** | 0.00006 | **23** | 0.00021 | **43** | 0.00073 | **63** | 0.00585 | **83** | 0.06230 |
| **4** | 0.00004 | **24** | 0.00022 | **44** | 0.00078 | **64** | 0.37835 | **84** | 0.06990 |
| **5** | 0.00004 | **25** | 0.00023 | **45** | 0.00088 | **65** | 0.00944 | **85** | 0.07789 |
| **6** | 0.00004 | **26** | 0.00025 | **46** | 0.00094 | **66** | 0.01040 | **86** | 0.08663 |
| **7** | 0.00003 | **27** | 0.00025 | **47** | 0.00102 | **67** | 0.01134 | **87** | 0.09606 |
| **8** | 0.00003 | **28** | 0.00027 | **48** | 0.00110 | **68** | 0.01267 | **88** | 0.10330 |
| **9** | 0.00004 | **29** | 0.00028 | **49** | 0.08412 | **69** | 0.01392 | **89** | 0.11421 |
| **10** | 0.00003 | **30** | 0.00030 | **50** | 0.00182 | **70** | 0.01519 | **90** | 0.12348 |
| **11** | 0.00004 | **31** | 0.00031 | **51** | 0.00195 | **71** | 0.01664 | **91** | 0.13686 |
| **12** | 0.00004 | **32** | 0.00034 | **52** | 0.00212 | **72** | 0.01850 | **92** | 0.15772 |
| **13** | 0.00005 | **33** | 0.00036 | **53** | 0.00238 | **73** | 0.02065 | **93** | 0.17384 |
| **14** | 0.00005 | **34** | 0.00040 | **54** | 0.00261 | **74** | 0.02280 | **94** | 0.19280 |
| **15** | 0.00007 | **35** | 0.00044 | **55** | 0.00288 | **75** | 0.02544 | **95** | 0.21337 |
| **16** | 0.00009 | **36** | 0.00043 | **56** | 0.00310 | **76** | 0.02853 | **96** | 0.23342 |
| **17** | 0.03190 | **37** | 0.00046 | **57** | 0.00332 | **77** | 0.03161 | **97** | 0.24805 |
| **18** | 0.00019 | **38** | 0.00050 | **58** | 0.00364 | **78** | 0.03513 | **98** | 0.28029 |
| **19** | 0.00020 | **39** | 0.00054 | **59** | 0.00399 | **79** | 0.03996 | **99** | 0.29939 |
| **20** | 0.00021 | **40** | 0.00060 | **60** | 0.00429 | **80** | 0.04497 | **100** | 0.32720 |

**Distribution of UK population by age and risk status**

| Age groups | Population distribution [9] | Distribution within age-group | | |
| --- | --- | --- | --- | --- |
|  |  | **Healthy** | **At risk** | |
|  |  |  | **Clinical at risk [5]** | **Residential care [8]** |
| **<5 years** | 6.20% | 93.96% | 6.04% | 0.00% |
| **5-17 years** | 14.91% | 93.96% | 6.04% | 0.00% |
| **18-49 years** | 44.16% | 92.48% | 7.52% | 0.00% |
| **50-64 years** | 18.19% | 83.41% | 16.59% | 0.00% |
| **65-69 years** | 4.71% | 52.05% | 42.95% | 5.00% |
| **70-74 years** | 3.96% | 52.05% | 42.95% | 5.00% |
| **75-79 years** | 3.22% | 52.05% | 42.95% | 5.00% |
| **80-84 years** | 2.40% | 52.05% | 42.95% | 5.00% |
| **>85 years** | 2.27% | 52.05% | 42.95% | 5.00% |

**All-cause mortality rates in the healthy population [4,6,7]**

| **Age** | Rate | **Age** | Rate | **Age** | Rate | **Age** | Rate | **Age** | Rate |
| --- | --- | --- | --- | --- | --- | --- | --- | --- | --- |
| **0** | 0.00308 |  |  |  |  |  |  |  |  |
| **1** | 0.00022 | **21** | 0.00026 | **41** | 0.00078 | **61** | 0.00319 | **81** | 0.01163 |
| **2** | 0.00013 | **22** | 0.00025 | **42** | 0.00084 | **62** | 0.00345 | **82** | 0.01290 |
| **3** | 0.00010 | **23** | 0.00026 | **43** | 0.00090 | **63** | 0.00392 | **83** | 0.01444 |
| **4** | 0.00008 | **24** | 0.00027 | **44** | 0.00096 | **64** | 0.00428 | **84** | 0.01620 |
| **5** | 0.00007 | **25** | 0.00028 | **45** | 0.00108 | **65** | 0.00219 | **85** | 0.01805 |
| **6** | 0.00007 | **26** | 0.00031 | **46** | 0.00115 | **66** | 0.00241 | **86** | 0.02007 |
| **7** | 0.00006 | **27** | 0.00030 | **47** | 0.00125 | **67** | 0.00263 | **87** | 0.02226 |
| **8** | 0.00006 | **28** | 0.00033 | **48** | 0.00135 | **68** | 0.00294 | **88** | 0.02394 |
| **9** | 0.00006 | **29** | 0.00034 | **49** | 0.00147 | **69** | 0.00322 | **89** | 0.02647 |
| **10** | 0.00006 | **30** | 0.00038 | **50** | 0.00122 | **70** | 0.00352 | **90** | 0.02861 |
| **11** | 0.00007 | **31** | 0.00039 | **51** | 0.00130 | **71** | 0.00385 | **91** | 0.03171 |
| **12** | 0.00007 | **32** | 0.00042 | **52** | 0.00142 | **72** | 0.00429 | **92** | 0.03655 |
| **13** | 0.00008 | **33** | 0.00045 | **53** | 0.00159 | **73** | 0.00479 | **93** | 0.04028 |
| **14** | 0.00009 | **34** | 0.00049 | **54** | 0.00175 | **74** | 0.00528 | **94** | 0.04468 |
| **15** | 0.00013 | **35** | 0.00054 | **55** | 0.00193 | **75** | 0.00590 | **95** | 0.04944 |
| **16** | 0.00017 | **36** | 0.00052 | **56** | 0.00208 | **76** | 0.00661 | **96** | 0.05409 |
| **17** | 0.00024 | **37** | 0.00056 | **57** | 0.00222 | **77** | 0.00732 | **97** | 0.05748 |
| **18** | 0.00024 | **38** | 0.00062 | **58** | 0.00244 | **78** | 0.00814 | **98** | 0.06495 |
| **19** | 0.00025 | **39** | 0.00067 | **59** | 0.00267 | **79** | 0.00926 | **99** | 0.06938 |
| **20** | 0.00025 | **40** | 0.00074 | **60** | 0.00287 | **80** | 0.01042 | **100** | 0.07582 |

**All-cause mortality rates in the at-risk population [4,6,7]**

| Age | Rate | Age | Rate | Age | Rate | Age | Rate | Age | Rate |
| --- | --- | --- | --- | --- | --- | --- | --- | --- | --- |
| **0** | 0.03085 |  |  |  |  |  |  |  |  |
| **1** | 0.00216 | **21** | 0.00256 | **41** | 0.00783 | **61** | 0.03189 | **81** | 0.11627 |
| **2** | 0.00133 | **22** | 0.00249 | **42** | 0.00837 | **62** | 0.03446 | **82** | 0.12899 |
| **3** | 0.00103 | **23** | 0.00259 | **43** | 0.00902 | **63** | 0.03921 | **83** | 0.14436 |
| **4** | 0.00078 | **24** | 0.00271 | **44** | 0.00962 | **64** | 0.04279 | **84** | 0.16197 |
| **5** | 0.00073 | **25** | 0.00280 | **45** | 0.01083 | **65** | 0.02187 | **85** | 0.18049 |
| **6** | 0.00066 | **26** | 0.00311 | **46** | 0.01153 | **66** | 0.02409 | **86** | 0.20075 |
| **7** | 0.00058 | **27** | 0.00303 | **47** | 0.01252 | **67** | 0.02627 | **87** | 0.22260 |
| **8** | 0.00064 | **28** | 0.00334 | **48** | 0.01354 | **68** | 0.02936 | **88** | 0.23938 |
| **9** | 0.00065 | **29** | 0.00345 | **49** | 0.01474 | **69** | 0.03225 | **89** | 0.26465 |
| **10** | 0.00059 | **30** | 0.00375 | **50** | 0.01217 | **70** | 0.03521 | **90** | 0.28613 |
| **11** | 0.00066 | **31** | 0.00386 | **51** | 0.01304 | **71** | 0.03855 | **91** | 0.31714 |
| **12** | 0.00071 | **32** | 0.00420 | **52** | 0.01418 | **72** | 0.04287 | **92** | 0.36548 |
| **13** | 0.00083 | **33** | 0.00446 | **53** | 0.01592 | **73** | 0.04785 | **93** | 0.40283 |
| **14** | 0.00094 | **34** | 0.00493 | **54** | 0.01749 | **74** | 0.05283 | **94** | 0.44676 |
| **15** | 0.00131 | **35** | 0.00536 | **55** | 0.01926 | **75** | 0.05896 | **95** | 0.49443 |
| **16** | 0.00168 | **36** | 0.00524 | **56** | 0.02076 | **76** | 0.06612 | **96** | 0.54088 |
| **17** | 0.00240 | **37** | 0.00563 | **57** | 0.02223 | **77** | 0.07324 | **97** | 0.57479 |
| **18** | 0.00238 | **38** | 0.00621 | **58** | 0.02438 | **78** | 0.08141 | **98** | 0.64949 |
| **19** | 0.00246 | **39** | 0.00667 | **59** | 0.02669 | **79** | 0.09260 | **99** | 0.69375 |
| **20** | 0.00253 | **40** | 0.00737 | **60** | 0.02874 | **80** | 0.10421 | **100** | 0.75820 |

**Sensitivity analysis parameters**

| **Variable** | **Base-case value** | **PSA distribution and 95% CI** | **Range for the one-way** |
| --- | --- | --- | --- |
| Circulation influenza A | 75.16% | Beta distribution  95% CI (56.44%;89.90%) | Range 29.70% to 99.60% |
| Matching within B | 52.36% | Beta distribution  95% CI (23.01%; 80.81%) | Range 0% to 99.00% |
| Vaccine efficacy TIV B perfect matching  0-4 yrs  5-17 yrs  18-49 yrs  50-64 yrs  65-69 yrs  70-74 yrs  75-79 yrs  80-84 yrs  85 to 100 yrs | 66.00%  77.00%  77.00%  73.00%  69.00%  69.00%  66.00%  66.00%  66.00% | Inverse Gamma  95%CI (12.32%;94.44%)  95%CI (19.42%;99.17%)  95%CI (19.42%;99.17%)  95%CI (17.08%;97.91%)  95%CI (14.45%;96.10%)  95%CI (14.45%;96.10%)  95%CI (12.32%;94.44%)  95%CI (12.32%;94.44%)  95%CI (12.32%;94.44%) | Range 44.00% to 94.44%  Range 52.00% to 99.17%  Range 52.00% to 99.17%  Range 49.00% to 97.91%  Range 47.00% to 96.10%  Range 47.00% to 96.10%  Range 44.00% to 94.44%  Range 44.00% to 94.44%  Range 44.00% to 94.44% |
| Vaccine efficacy TIV B absence of matching  0-4 yrs  5-17 yrs  18-49 yrs  50-64 yrs  65-69 yrs  70-74 yrs  75-79 yrs  80-84 yrs  85 to 100 yrs | 44.00%  52.00%  52.00%  49.00%  47.00%  47.00%  44.00%  44.00%  44.00% | Linked to the Inverse Gamma distribution of the VE TIV B perfect matching  95%CI (1.63%;94.44%)  95%CI (1.52%;99.17%)  95%CI (1.52%;99.17%)  95%CI (1.56%;97.91%)  95%CI (1.58%;96.10%)  95%CI (1.58%;96.10%)  95%CI (1.63%;94.44%)  95%CI (1.63%;94.44%)  95%CI (1.63%;94.44%) | Range 15.38% to 66.00%  Range 20.61% to 77.00%  Range 20.61% to 77.00%  Range 18.60% to 73.00%  Range 17.61% to 69.00%  Range 17.61% to 69.00%  Range 15.38% to 66.00%  Range 15.38% to 66.00%  Range 15.38% to 66.00% |
| Cost of GSK-QIV | £6.72 | Not applicable | Range £6.72 to £10.08 (+50%) |
| Influenza duration | 7.50 days | Gamma distribution  95% CI (0.19;27.67) | 95% CI (0.19 to 27.67) |
| Probability of influenza A unvaccinated | Children: 19.21%  Adults: 6.55%  Elderly: 6.17% | Lognormal distribution  Children : 95% CI (14.99%;24.63%)  Adults : 95% CI (3.15%;13.62%)  Elderly : 95% CI (2.97%;12.83%) | 95% CI |
| Probability of influenza B unvaccinated | Children: 19.21%  Adults: 6.55%  Elderly: 6.17% | Lognormal distribution  Children : 95% CI (14.99%;24.63%)  Adults : 95% CI (3.15%;13.62%)  Elderly : 95% CI (2.97%;12.83%) | 95% CI |

# References
